# Case report of laparoscopic reduction of retro-ureter incarcerated small bowel obstruction

**DOI:** 10.1097/MD.0000000000018250

**Published:** 2019-12-10

**Authors:** Sungwoo Cho, Sangchul Yun, Yunhee Lee, Myong Hoon Ihn

**Affiliations:** Department of Surgery, Soonchunhyang University Seoul Hospital, Seoul, South Korea.

**Keywords:** internal hernia, intestinal obstruction, ureter

## Abstract

**Rationale:**

Various types of internal hernias have been reported including paraduodenal, intersigmoidal, pericecal, foramen of Winslow, as well as transmesenteric and retroanastomotic hernias. However, small bowel obstruction secondary to an internal hernia caused by the ureter is rare, and only a few cases have been reported worldwide. We report a case of small bowel herniation caused by the ureter in a woman who underwent radical hysterectomy for cervical cancer.

**Patient concerns:**

A 53-year-old woman presented with acute abdominal pain and vomiting and reported a history of radical hysterectomy for cervical cancer 6 years prior to presentation.

**Diagnoses:**

Computed tomography revealed segmental luminal dilatation of pelvic ileal loops, 2 transition zones with the beak sign in the left-sided pelvic cavity, and reduced enhancement of bowel loops. Hydronephrosis with abrupt luminal narrowing of the left distal ureter was also observed.

**Interventions:**

Exploratory laparoscopy revealed incarcerated bowel segments beneath an adhesive band. We did not immediately cut the adhesive band and continued to trace the course of the small bowel and attempted reduction of the hernia. Reduction of the hernia was not difficult; therefore, the entire small bowel could be disentangled from the pelvic adhesions without any small bowel injury. After reduction of the herniated small bowel, we could confirm that the adhesive band was the left ureter (ureteral peristalsis was observed). The reduced segments of the small bowel appeared viable, and resection was not required.

**Outcomes:**

The patient was discharged 2 days postoperatively without any complication.

**Lessons:**

Cutting band during adhesiolysis enables release of bowel obstruction. However, owing to the different types of internal hernias that are known to occur, it is essential to confirm the patient's history and preoperative CT findings to avoid complications.

## 1 Introduction

Various types of internal hernias have been reported in the literature. Any site of potential weakness or defect (usually in the abdominal wall) through which the viscera (or part of the viscera) can protrude can cause internal herniation. Common types of internal hernias was reported in the literature.^[1]^ However, cases of internal hernia in the pelvic cavity are rare. The literature contains a few case reports of herniation through the pouch of Douglas,^[2]^ through a congenital peritoneal defect,^[3]^ or through a defect in the broad ligament.^[4]^ However, retro-ureteral small bowel incarceration is extremely rare, and only a few cases have been reported worldwide.^[5–10]^ We report a case of retro-ureteral small bowel obstruction in a woman who underwent radical hysterectomy for cervical cancer. We would like to say what is the way to avoid the complications that may occur during intestinal obstruction surgery.

## 2 Case presentation

**Figure 1:**
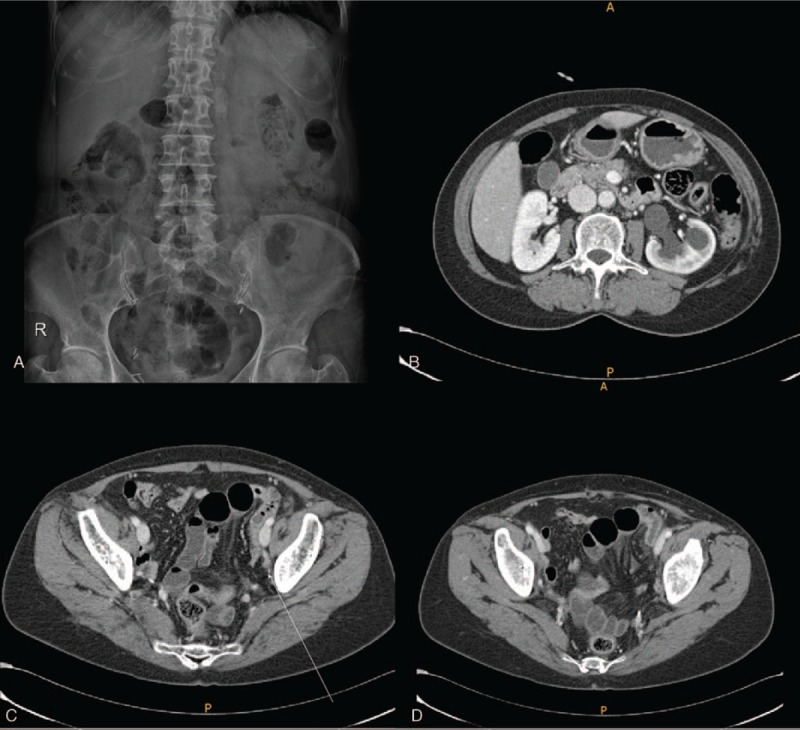
Plain radiography and computed tomography findings. A. Plain radiographs showing a non-specific bowel gas pattern. B. Left-sided hydronephrosis is observed. C. Arrow indicates abrupt luminal narrowing of the left distal ureter secondary to a suspected left distal ureteral stricture. D. CT scan showing segmental luminal dilatation of the pelvic ileal loops and 2 transition zones with the beak sign in the left-sided pelvic cavity. Reduced enhancement of the bowel loops is visualized. Mesenteric congestion is observed, and fluid collection is visualized in the pelvic ileal loops. Findings are suggestive of suspected closed-loop obstruction of pelvic ileal loops with strangulation secondary to an adhesive band. The CT scan additionally shows a post-hysterectomy state.

**Figure 2:**
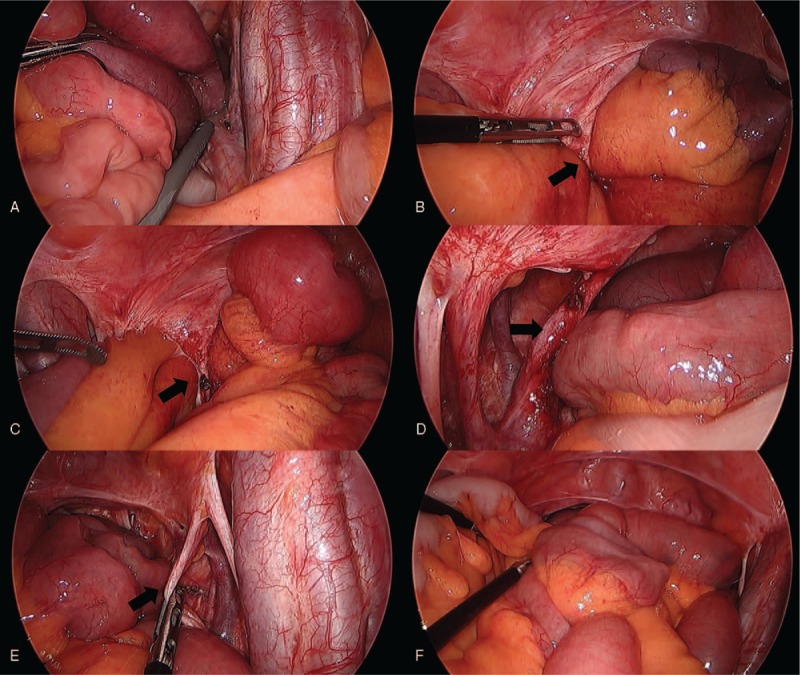
Intraoperative laparoscopic findings. A. Intraoperative laparoscopic images showing segmental small bowel ischemic changes in the pelvic area. It was difficult to trace the ileum in the pelvic area. B. The small bowel was traced from the proximal part to the pelvis. The adhesive band (which was the cause of strangulation) can be observed in the pelvic area (arrow). C. The adhesive band is not immediately cut, and the small bowel is traced further. The herniated small bowel was reduced and separated from the adhesive band without any bowel injury. D. Image showing the adhesive band (left ureter) (arrow). Ureteral peristalsis was confirmed. E. Image showing the right ureter detached from the surrounding structures (arrow). F. Image showing the reduced small bowel segment without any ischemic changes.

Patient has provided informed consent for publication of the case. A 53-year-old woman presented with acute abdominal pain, vomiting, and a 2-day history of constipation. She reported a history of undergoing an operation for cervical cancer at another hospital, 6 years prior to presentation. She also underwent an operation for ureteral stricture 25 years earlier. However, details regarding the type of surgery were unavailable. Her medication history included the use of medication for rheumatoid arthritis. Physical examination showed an acutely ill-looking woman. Systolic blood pressure was 125 mm Hg, pulse rate was 71 beats/minute, and body temperature was 36.8°C. Systemic examination revealed diffuse abdominal tenderness predominantly in the lower abdomen. Plain abdominal radiographs revealed a non-specific bowel gas pattern (Fig. 1A). However, computed tomography (CT) revealed segmental luminal dilatation of the pelvic ileal loops, 2 transition zones with the beak sign observed in the left-sided pelvic cavity, and reduced enhancement of bowel loops. Mesenteric congestion was observed, and the pelvic ileal loops showed fluid collection. Closed-loop obstruction of pelvic ileal loops with adhesive band-induced strangulation was suspected (Fig. 1C, D). Additionally, left-sided hydronephrosis was observed with abrupt luminal narrowing of the left distal ureter (suspected to be secondary to a stricture in the left distal ureter) (Fig. 1B) CT revealed an absent uterus (post-hysterectomy state). The patient's white blood cell count, as well as serum C-reactive protein and lactic acid levels were slightly increased to 12100 cells/μl (4000–10000/μl), 3.16 mg/dl (0–0.5 mg/dl), and 2.7 mmol/L (0.5–2.2 mmol/L), respectively. Serum blood urea nitrogen (BUN) and creatinine levels, as well as the estimated glomerular filtration rate were within reference range. Emergency laparoscopy was performed using 1 11-mm and 2 5-mm trocars. Abdominal inspection during the laparoscopy showed segmental small bowel ischemic changes in the pelvis (Fig. 2A). We traced the course of the small bowel from the ileocecal valve. The course of the ileum could not be traced in the vicinity of the pelvis, and we deduced that using a greater degree of traction could injure the strangulated small bowel (Fig. 2B). Laparoscopic exploration was continued, and we traced the course of the proximal segments of the small bowel, and the jejunum was traced distally to the pelvis. When exploration continued in the vicinity of the pelvis, we could observe a fibrotic adhesive band, which had caused the internal herniation (Fig. 2C). However, immediate adhesiolysis was not performed, and we continued to trace the small bowel and attempted reduction. Reduction of the hernia was not difficult; therefore, the entire small bowel could be disentangled from the pelvic adhesion without any small bowel injury. Finally, we confirmed that the adhesive band was the left ureter (based on definitive evidence of ureteral peristalsis) (Fig. 2D). We concluded that the ureter had been skeletonized during the hysterectomy performed for cervical cancer. Both ureters were completely detached from the surrounding structures (Fig. 2E). We elected not to repair the retro-ureteral space because normal ureteral peristalsis was observed bilaterally, and a suture or mesh could interfere with ureteral function and/or cause ureteral stricture. The reduced small bowel segment did not show ischemia and was therefore not resected (Fig. 2F). The patient was discharged 2 days postoperatively without any complications. Follow-up period was 16 months. The patient's serum BUN and creatinine levels were within reference range, and she did not report any urinary discomfort. Therefore, she refused any further tests including ultrasonography to assess hydronephrosis.

## 3 Discussion

Radical hysterectomy and pelvic lymph node dissection are standard operations to treat cervical cancer.^[11]^ Extensive dissection and intra-abdominal adhesions tend to cause postoperative intestinal obstruction. However, internal herniation rarely causes intestinal obstruction in patients undergoing pelvic surgery. A broad ligament hernia is a known type of internal hernia observed in women undergoing pelvic surgery.^[4]^ Although extremely rare, a ureteric band can cause internal hernia in patients undergoing pelvic operations, such as ureteral reimplantation or radical hysterectomy. Our patient had undergone radical hysterectomy and showed retro-ureteral small bowel herniation. This is the first case report that describes laparoscopic reduction of internal hernia secondary to prolapsed small bowel under an adhesive ureteral band in a woman with a history of radical hysterectomy. Similar herniation and bowel obstruction can occur after pelvic surgeries involving ureteral mobilization.

Our literature search revealed only 9 patients of retro-ureteral internal hernias, with our patient being the 10th reported case. Of these 10 patients, a radical hysterectomy was performed in 4 patients,^[10,12,13]^ a radical cystectomy and ileal conduit in 3,^[5,9,14]^ and ureteral reimplantation in 3 patients^[6–8]^ (Table 1). The mean age of the 10 patients was 54.3 years, and these patients included 6 women and 4 men. The right ureter was involved in 4 and the left ureter in 6 patients. The time interval between the initial operation and the time of presentation varied between 12 days and 20 years. In 3 of the reported cases,^[8,12,13]^ the ureteral band could not be identified before it was cut to release the incarcerated small bowel. It is important to identify this structure preoperatively to release the incarcerated small bowel without ureteral division. The adhesive band was suspected to be the ureter using preoperative CT in only 2 cases. Ataka et al^[9]^ reported a study in which CT revealed intestinal dilatation with a band crossing the pelvis, which was suspected to be the displaced ureter suggesting intestinal obstruction secondary to internal herniation. Flores et al^[6]^ reported a study in which CT showed small bowel strangulation with the stretched ureter bridging the mesentery of the involved small bowel loops anteriorly. These findings indicated strangulated small bowel obstruction secondary to retro-ureteral small bowel hernia. Flores et al identified the following CT features that suggest ureteral involvement as a cause of an internal hernia:

1. cluster of strangulated small bowel loops located in the right flank area,
2. adjacent beak signs with 2 transition zones of the closed loop,
3. stretching of the ureter from the kidney to the bladder and,
4. the mesentery of the herniated small bowel crossing posterior to the ureteric band.^[6]^

**Table 1:**
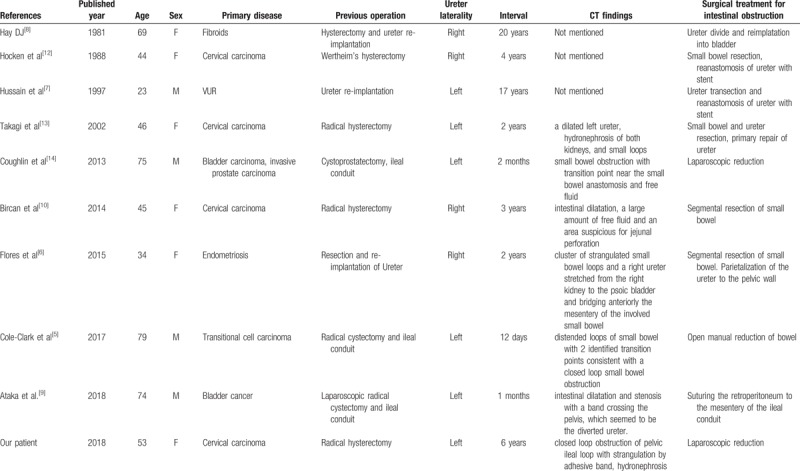
Currently reported patients with retro-ureteric small bowel obstruction.

In this patient, we performed CT to confirm the site of obstruction and/or recurrence of the condition. CT suggested closed-loop obstruction of the pelvic ileal loops with strangulation caused by an adhesive band; however, CT could not conclusively determine that the adhesive band was the left ureter. It is commonly accepted that if radiological imaging does not reveal a mass, an adhesive band could be tentatively considered the cause of intestinal obstruction. In this patient, small bowel obstruction was attributed to an adhesive band, and we performed emergency surgery. Previous dissection of the soft tissue in the area led to exposure of the ureter and enabled incarceration of the small bowel in the left retro-ureteral space. After releasing the small bowel obstruction, the stretched and narrowed ureter showed re-expansion. Retrospective CT review revealed hydronephrosis with a dilated left ureter, which showed abrupt narrowing at the left-sided pelvic inlet.

Minimally invasive surgeries such as laparoscopy and robotic surgery may cause exposure of areas of weakness/defects that predispose to herniation. There is lack of data regarding the outcomes of mesenteric closure and postoperative bowel obstruction caused by internal hernia. Mesenteric closure to reduce the risk of internal herniation remains debatable. A few authors recommend closure of all potential spaces,^[15–17]^ whereas a few others report no difference in outcomes between patients with and without closure.^[18–20]^ Interestingly, it has been reported that defect closure could increase potential complications.^[21,22]^ It has also been suggested that the size of the defect may be an important determinant of internal herniation in that a large defect is unlikely to trap and obstruct bowel loops.^[1]^

In this patient, after laparoscopic reduction of the incarcerated small bowel, we consulted a senior surgeon and elected not to close the defect. Owing to extensive dissection during the radical hysterectomy that was performed previously, there was lack of soft tissue to close the space. The retro-ureteral space is a relatively large space that allows unrestricted mobility of the small bowel through it. Laparoscopic reduction of the herniated small bowel under ureteral bands is relatively easy. The small bowel obstruction was released without any bowel injury, and we did not need to cut the band. The effects of fixing the ureter to the pelvic wall or application of a mesh to cover the pelvis including the ureter remain unclear. Non-closure of the retro-ureteral space can cause recurrent hernia. However, this patient has not reported any obstructive symptoms for 10 months after laparoscopic reduction.

## 4 Conclusion

Extensive ureteral mobilization performed during radical hysterectomy with pelvic lymphadenectomy without reperitonealizing the ureters exposed the ureters after removal of fatty tissue. This allowed the bowel to undergo incarceration in the retro-ureteral space. Preoperative CT diagnosis of this rare type of hernia is important because the stretched intraperitoneal ureter may resemble an adhesive band, particularly in patients with a history of pelvic operations. Laparoscopic reduction of the herniated small bowel under ureteral bands is relatively easy. The small bowel obstruction could be released without any bowel injury, and we can avoid incidental ureter injury.

## Author contributions

**Conceptualization:** Sangchul Yun, Yunhee Lee.

**Project administration:** Sangchul Yun.

**Supervision:** Sangchul Yun, Myong Hoon Ihn.

**Writing – original draft:** Sungwoo Cho.

**Writing – review & editing:** Sangchul Yun, Myong Hoon Ihn.

Sangchul Yun orcid: 0000-0002-6321-4319.
